# Editorial: Mental health of higher education students

**DOI:** 10.3389/fpsyt.2022.1089877

**Published:** 2022-12-06

**Authors:** Agnes Yuen-Kwan Lai, Wing-Fai Yeung

**Affiliations:** ^1^Li Ka Shing Faculty of Medicine, School of Nursing, The University of Hong Kong, Pokfulam, Hong Kong SAR, China; ^2^Faculty of Health and Social Sciences, School of Nursing, The Hong Kong Polytechnic University, Kowloon, Hong Kong SAR, China

**Keywords:** university, higher education, students, depression, anxiety, mental health, support

Mental health of higher education students is a growing concern around the globe. Even before the outbreak of the COVID-19 pandemic, the prevalence of mental health problems among higher education students was already worrying. The Healthy Minds Network, a US national research organization, showed that depression and anxiety were found in 37.1 and 31.3% of higher education students, respectively (1). Higher education students encounter transitional challenges and stressors while preparing themselves to enter adulthood. Besides academic burdens, university students struggle to fulfill expectations from family, adapt to a drastic change in the learning environment, develop good relationships with peers, and overcome financial problems. The unprecedented wave of COVID-19 further worsened the already serious problems. Many students experienced psychological distress during the pandemic, including anxiety, stress, and even suicidal ideation (2–5). This Research Topic attempts to gather empirical findings from all over the world to broaden our understanding of the mental health of higher education students.

This Research Topic collects 34 studies on the mental health of higher education students, with more than 120,000 participants from 18 countries, among nationwide (Gavurova et al.; Jiang et al.; Qian et al.; Wathelet et al.). Studies in the Research Topic investigated a wide range of mental health outcomes, from symptom-related outcomes such as anxiety, depression, and stress (Schwander-Maire et al.; Thang et al.; Valdés et al.; Wathelet et al.; Ying et al.), insomnia (Stanyte et al.; Zhang et al.), to behavioral problems such as internet addiction (Diotaiuti et al.; Gavurova et al.), mobile phone addiction (Li X. et al.), school engagement (Versteeg et al.), academic performance (AbuAlSamen and El-Elimat; Deng et al.), and problem gaming behavior (Tang et al.).

A vast array of factors associated with the outcomes were examined, including hope (Schwander-Maire et al.), impulsivity (Diotaiuti et al.), family functioning (Qian et al.), resilience (Versteeg et al.), shyness (Li X. et al.), cyberloafing (Li Q. et al.). Three studies provided evidence with quantitative analysis to validate the instruments in their populations (AbuAlSamen and El-Elimat; Fung et al.; Guelmami et al.). Findings in this Research Topic help aggregate knowledge in literature for mental health promotion of higher education students and suggest actionable strategies for future interventions and policies.

## Author contributions

Both authors listed have made a substantial, direct, and intellectual contribution to the work and approved it for publication.
